# Childhood obesity in changing food systems: mediterranean diet, socioeconomic inequalities and AI-driven personalized nutrition — the case of Italy

**DOI:** 10.3389/fpubh.2026.1883078

**Published:** 2026-09-17

**Authors:** Maria Teresa Montagna, Giuseppina Caggiano, Osvalda De Giglio, Rachele Maria Russo, Francesco Triggiano, Stefano Orlando, Anna Maria Doro Altan, Michele Fernando Panunzio

**Affiliations:** 1 Interdisciplinary Department of Medicine, Hygiene Unit, University of Bari Aldo Moro, Bari, Italy; 2 Local Health Authority, Foggia, Italy; 3 Department of Biomedicine and Prevention, University of Rome “Tor Vergata”, Rome, Italy; 4 Department of Life Sciences, Health and Health Professions, Link Campus University, Rome, Italy

**Keywords:** artificial intelligence, childhood obesity, food systems, health equity, mediterranean diet, personalized nutrition, socioeconomic inequalities

## Abstract

Childhood obesity is a major global public health challenge resulting from the interaction between changing food systems, dietary behaviors, and social inequalities. This Perspective argues that childhood obesity should be addressed through an integrated systems-based framework that links food environments, socioeconomic determinants, and emerging digital health technologies.

Across Europe, children’s dietary patterns have shifted toward increased consumption of ultra-processed foods and energy-dense products, alongside low adherence to healthy dietary guidelines. These trends disproportionately affect socially disadvantaged populations, reflecting persistent socioeconomic inequalities. Italy provides an informative case in which high levels of childhood overweight and obesity coexist with the cultural heritage of the Mediterranean diet, illustrating the gap between traditional dietary patterns and modern obesogenic environments. Although the Mediterranean diet remains a key model for healthy and sustainable eating, adherence is declining among younger generations, which points to the need for structural and educational interventions. Artificial intelligence (AI) and digital health technologies are being investigated as tools to support surveillance, early risk detection, and personalized nutrition strategies. However, evidence of sustained effectiveness in childhood obesity prevention remains limited, and any benefit will depend on equitable access, robust governance, and integration within existing health and education systems. Preventing childhood obesity therefore requires moving beyond exclusively individual-level interventions toward coordinated, multisectoral strategies that address food environments, social determinants of health, and equity. The combination of sustainable dietary models such as the Mediterranean diet, school- and community-based interventions, and responsibly implemented digital innovations may contribute to more effective and equitable prevention strategies.

## Introduction

Over the past few decades, dietary habits among children and adolescents have changed profoundly worldwide, with serious implications for public health. One of the most significant epidemiological trends has been the global increase in overweight and obesity. According to recent reports from UNICEF and the World Health Organization (WHO), obesity is expected to surpass underweight as the most prevalent form of malnutrition among school-age children and adolescents (5–19 years) (1, 2). This phenomenon is part of the broader ‘nutrition transition’, characterized by changes in food systems, dietary patterns, and lifestyle behaviors. Initially observed mainly in high-income countries, childhood obesity is now increasingly prevalent in middle- and low-income countries, reflecting the global spread of energy-dense, nutrient-poor diets (3).

Environmental changes are also contributing to the transformation of children’s dietary behaviors. Climate change and the globalization of food systems influence the availability and quality of food, reducing access to fresh, nutrient-dense foods and increasing reliance on ultra-processed foods (UPFs) (4, 5). At the same time, commercial pressures, socioeconomic inequalities, and environments that promote obesity are reshaping dietary habits and health risks among younger populations. Meanwhile, digital and data-driven technologies are emerging as new tools for monitoring dietary behaviors and supporting prevention strategies, including AI-driven personalized nutrition approaches.

This Perspective examines childhood obesity as a complex, multifactorial condition that is influenced by food systems, socioeconomic inequalities, dietary transitions, and cultural factors. An interdisciplinary and systems-oriented perspective was adopted to critically integrate international epidemiological, nutritional and public health evidence. Italy is a particularly informative case study because it brings together, within a single national context, a strong Mediterranean dietary heritage, persistently high childhood overweight and obesity, marked socioeconomic and geographical inequalities, changing food environments and emerging inequalities in digital access. This combination provides an opportunity to examine how cultural dietary assets interact with contemporary structural determinants and how digital innovation might complement, rather than substitute for, population-level prevention. By contextualizing the Italian experience within broader European trends, this Perspective highlights the need for integrated prevention strategies that combine sustainable dietary models, public health nutrition policies, and emerging digital approaches. The broader lesson is not that the Italian experience can be directly generalized to all European or Mediterranean countries, but that similar settings may face a common implementation challenge: healthy dietary traditions can coexist with contemporary food environments that make adherence increasingly difficult and socially patterned. The Italian experience therefore supports a transferable principle rather than a universally transferable model—childhood obesity prevention should align dietary recommendations with interventions addressing affordability, food environments and socioeconomic inequalities, while ensuring that digital innovations do not create additional barriers for disadvantaged populations.

The novel conceptual contribution of this Perspective is an equity-oriented, multilevel framework that situates Mediterranean diet adherence and AI-enabled personalized nutrition within the food-system and socioeconomic conditions that shape childhood obesity. Rather than treating dietary behavior, social inequalities and digital innovation as separate domains, the framework considers AI as a cross-cutting support for individual-, school/community- and population-level prevention, while recognizing that technological interventions cannot substitute for action on structural determinants of health.

### Materials and methods: literature identification

For the artificial intelligence (AI)-related sections of this Perspective, we used a targeted, non-systematic literature-identification approach designed to support conceptual synthesis rather than exhaustive evidence retrieval. PubMed/MEDLINE and Google Scholar were searched during manuscript development and updated on 4 August 2026. No formal start date was imposed; priority was given to publications from 2020 onwards, while earlier seminal studies were retained when directly relevant. Search terms were combined iteratively and included (“artificial intelligence” OR “machine learning” OR “deep learning” OR “digital health” OR wearable* OR “continuous monitoring”) AND (“childhood obesity” OR “paediatric obesity” OR child* OR adolescen*) AND (“personalized nutrition” OR “precision nutrition” OR “dietary assessment” OR “risk prediction” OR “food marketing”), together with “social determinants of health”, “socioeconomic inequalities”, “health equity”, “algorithmic bias” and “digital divide.” Searches were complemented by AI-assisted literature discovery using ChatGPT Deep Research and Semantic Scholar to accelerate candidate identification, citation tracing and prioritisation. Recent systematic reviews, umbrella reviews and meta-analyses were prioritised, together with methodologically informative primary studies and authoritative reports from public health institutions. Selection was purposive and based on direct relevance to the argument, study design and methodological quality, recency, and scientific influence within the field. Reference lists of key sources were checked, and every AI-suggested record was verified against the original publication before inclusion. Consistent with the aims of a Perspective article, this process did not involve exhaustive retrieval, duplicate independent screening, PRISMA-based study selection or formal risk-of-bias grading.

### European trends in childhood nutrition and obesity

In response to the growing obesity epidemic, the countries of the WHO European Region adopted the European Charter on Counteracting Obesity in 2006. This charter outlines guiding principles and priority actions at local, national, and international levels.

According to the most recent data from the Childhood Obesity Surveillance Initiative (COSI), around 25% of 6–9-year-old children in Europe are overweight or obese, with 11% specifically affected by obesity (1, 6). Only 42.5% of children consume fruit daily and 22.6% consume vegetables daily, although there is marked variation across countries. Higher prevalence rates are consistently observed in southern European countries. Meanwhile, levels appear stable or are increasing slightly in northern and eastern Europe (7). Children’s food preferences are further influenced by exposure to digital food marketing, while socioeconomic inequalities restrict access to healthier dietary options. Taken as a whole, these patterns illustrate the interaction between food systems, commercial influences, social inequalities, and public health policies.

The Health Behavior in School-aged Children (HBSC) survey (adolescents 11–15 years) documents pronounced socioeconomic and behavioral inequalities (8, 9). Despite some regional variation, progress in reducing childhood overweight remains limited. In many countries, dietary patterns are increasingly characterized by high consumption of ultra-processed foods and energy-dense drinks, which replace fresh, nutrient-rich foods (2, 3).

Viewed together, the European data depict childhood obesity as arising from an interplay of dietary, environmental and structural factors within an increasingly ‘obesogenic’ food environment. This evidence supports efforts to improve dietary quality and the food environment during the early years of life.

These surveillance needs provide a rationale for evaluating digital monitoring systems and AI-supported public health interventions.

### Childhood obesity in Italy: epidemiological profile and social inequalities

In Europe, Italy is an example of the so-called ‘Mediterranean paradox’, where traditional healthy dietary patterns coexist with a relatively high prevalence of childhood obesity, particularly among children from socio-economically disadvantaged backgrounds.

OKkio alla SALUTE is a national surveillance system that monitors overweight and obesity among third-grade primary school children, as well as related behavioral risk factors. It forms part of the COSI program and is coordinated by the Italian National Institute of Health (ISS), in collaboration with the Ministry of Education and regional health authorities (10, 11). According to the most recent data, 19% of Italian children aged 8–9 are overweight, 9.8% are obese, and 2.6% are severely obese (12). Although the prevalence of being overweight has declined progressively since 2008 (from 23.2 to 20.9% in 2014), the prevalence of obesity has remained relatively stable since then, fluctuating around 9–10%. A persistent north–south gradient is evident, with southern and some central regions having higher rates than the national average (13).

Socioeconomic disparities are also evident in dietary and lifestyle behaviors. Children from lower socioeconomic status (SES) families consume fewer fruits and vegetables, report higher intake of sugar-sweetened beverages and UPFs, and are more likely to engage in sedentary behaviors. In southern Italy, higher levels of physical inactivity, irregular breakfast consumption and screen exposure (e.g., having a television in the bedroom) have been reported (12, 14–16). These patterns are consistent with structural inequalities in income, education, and access to health-promoting resources. At national level, surveillance data indicate that childhood obesity in Italy is a multifactorial condition strongly influenced by household environments and broader social determinants of health.

Evidence from the PASSI (adults aged 18–69) and PASSI d’Argento (adults aged ≥65) surveillance systems indicates that obesity-related risk factors persist throughout life, which supports attention to prevention early in life (17). The strategies considered in this Perspective include promoting the Mediterranean diet in schools and communities, alongside family-based interventions and tailored public health policies. Approaches guided by the principle of proportionate universalism and aligned with European equity-oriented frameworks may be especially relevant. Italy is therefore informative not simply because of its relatively high prevalence of childhood obesity, but because several components of the proposed framework coexist within the same national context. A strong Mediterranean dietary heritage coexists with declining adherence to traditional dietary patterns and increasing exposure to obesogenic food environments; marked socioeconomic inequalities intersect with a persistent North–South gradient in childhood obesity and health-related behaviors; and emerging digital-health opportunities coexist with geographical and socioeconomic inequalities in digital access. The Italian case therefore illustrates how the availability of a culturally established healthy dietary model does not in itself protect children from obesity when structural, commercial and socioeconomic conditions constrain its adoption. It also shows why digital and AI-enabled approaches need to be embedded within, rather than considered separately from, food-environment and equity-oriented policies. Table 1 summarizes data from the COSI, the HBSC survey, and national surveillance data from the ISS and the Ministry of Health. This allows comparison across the pediatric age spectrum in both European and Italian contexts.

**Table 1:** Prevalence of overweight and obesity, dietary behaviors, and socioeconomic disparities among children and adolescents in Europe and Italy (2022–2025).

Indicator	Europe (COSI/HBSC)	Italy
Overweight + obesity prevalence	~20–25% (children/adolescents)	28.8% (children), ~20–25% (adolescents)
Obesity prevalence	~5–11%	9.8% (2.6% severe)
Dietary behavior	Suboptimal diet quality, high UPF consumption	Low adherence to Mediterranean diet, high UPF intake
Physical activity	Generally low to moderate	Low levels
Socioeconomic inequalities	Strong gradient across countries	Strong gradient, higher risk in low SES
Geographical disparities	Present	North–South gradient

### The Mediterranean diet as sustainable nutritional model

The Mediterranean diet (MD), which originates from countries bordering the Mediterranean Sea such as Italy, Greece and Spain, is widely recognized as a healthy and sustainable dietary pattern (18). Evidence from prospective cohort studies and meta-analyses, derived predominantly from adult populations, associates adherence to the MD with lower mortality and a reduced incidence of major chronic diseases (19–21). Evidence specifically concerning childhood and adolescent obesity is less extensive and more heterogeneous. Observational studies have reported associations between greater MD adherence and more favourable BMI or body-composition profiles in adolescents (22, 23); however, findings regarding weight status have not been fully consistent across pediatric studies (24), and observational associations should not be interpreted as evidence that MD adherence independently prevents childhood obesity.

The MD is characterized nutritionally by high consumption of fruits, vegetables, legumes and whole grains; moderate intake of fish and dairy products; and olive oil as the main source of fat. Consumption of red meat and refined sugars is limited (25). Beyond its nutritional profile, the MD represents a broader cultural and environmental framework integrating diet, lifestyle, sustainability and social interaction. This is particularly relevant during childhood, when eating habits are formed (26).

The principles of the MD have also been adapted in non-Mediterranean contexts. The Nordic diet, for instance, emphasizes whole grains, oily fish, legumes, berries, and unsaturated vegetable oils, reflecting the MD’s emphasis on minimally processed, nutrient-dense foods (27). Similarly, Brazilian dietary guidelines promote fresh, minimally processed foods while discouraging ultra-processed products (28). These examples suggest that the principles of Mediterranean dietary patterns can be adapted beyond their regions of origin.

Despite its recognized benefits, adherence to the MD is declining in its countries of origin. In Italy, for example, data from the OKkio alla Salute surveillance system shows that 25% of children do not eat fruit or vegetables every day, 24.6% drink sugar-sweetened beverages daily, and 53% eat snacks at least three times a week (12). The pediatric evidence also presents important methodological limitations. Much of the literature examining the relationship between MD adherence and weight status is observational and frequently cross-sectional, limiting causal inference and leaving the possibility of residual confounding and reverse causation (24). Comparability across studies is further complicated by heterogeneity in the assessment of MD adherence. Although the KIDMED index is the most widely used scoring system in children and adolescents, differences in adherence indices, thresholds and dietary assessment methods limit comparability across studies, and further validation of pediatric MD indices has been recommended (24). Intervention evidence is more encouraging but remains heterogeneous. A meta-analysis of randomized controlled trials found a small but statistically significant reduction in BMI following MD-based interventions in children and adolescents (Cohen’s d = −0.14), with substantial between-study heterogeneity (I^2^ = 77.5%) (29). More recent evidence focusing specifically on children and adolescents with overweight or obesity also suggests potential anthropometric benefits of MD-based dietary interventions, although high study heterogeneity and non-significant findings for some outcomes indicate that more robust evidence is still needed (30). Overall, current evidence supports the MD as a potentially favourable component of pediatric obesity prevention and management but does not establish MD adherence alone as a consistently effective strategy for preventing childhood obesity.

Importantly, adherence to the MD should not be interpreted solely as an individual dietary choice. The ability of children and families to maintain a Mediterranean dietary pattern is embedded within broader socioeconomic and food-environment conditions, including food availability and affordability, household resources, time available for food preparation, nutritional literacy and access to health-promoting environments. Socioeconomic position may therefore influence the accessibility and sustainability of MD adherence, although pediatric studies have not shown a fully consistent association between socioeconomic status and MD adherence (24). This uncertainty reinforces the need to avoid attributing low adherence exclusively to individual behavior and to consider the structural conditions under which dietary choices are made. Promoting the MD as part of childhood obesity prevention consequently requires not only nutritional education but also measures that make healthy dietary choices accessible, affordable and sustainable across socioeconomic groups.

The updated Mediterranean food pyramid (31) provides an integrated framework combining the nutritional, cultural, and environmental dimensions of the MD. As well as making recommendations about food groups, it emphasizes seasonality, local food production, physical activity, adequate rest and social relationships. This framework is intended to address contemporary challenges such as climate change, biodiversity loss and the increase in non-communicable diseases.

Although the MD has been recognized by UNESCO as part of Italy’s intangible cultural heritage and as a global public health model, it is facing increasing challenges due to the progressive adoption of Westernized dietary patterns among younger generations.

Rather than implying a single established causal pathway, we propose an integrative conceptual framework linking structural, dietary and technological dimensions of childhood obesity prevention. Within this framework, children’s capacity to adhere to healthy dietary patterns such as the Mediterranean diet is shaped by interacting food-system and socioeconomic conditions, including food availability and affordability, exposure to marketing, family and school environments, neighborhood infrastructure and socioeconomic resources. AI represents a cross-cutting rather than independent component of this framework: it may support individualized risk assessment and tailored nutritional support, while also integrating behavioral, social and environmental data for population surveillance and policy planning (32). Its potential contribution should therefore be evaluated in relation to, rather than separately from, the structural conditions that shape childhood obesity.

### Emerging role of artificial intelligence in public health

Artificial intelligence (AI) is increasingly being investigated as a potential tool for understanding childhood obesity and supporting prevention. Applications have been explored in infectious-disease surveillance, including waterborne diseases, and in the prevention and management of chronic diseases (33–36); however, experience in these areas should not be assumed to establish effectiveness in pediatric obesity.

One key application is AI-driven personalized nutrition, which uses omics data (e.g., genomics and metabolomics), dietary intake, and biomarker information to improve on traditional ‘one-size-fits-all’ dietary recommendations (37, 38). Such approaches may support adherence to healthy dietary patterns, including the Mediterranean diet, but evidence that they produce sustained dietary change or prevent non-communicable diseases in children remains limited. Their ability to combine biological, behavioral and social data could support earlier and more targeted prevention, although their added value over validated nutritional assessment and established public health interventions has yet to be demonstrated.

Machine learning and computer vision techniques may facilitate the monitoring of dietary behaviors and the earlier identification of unhealthy patterns (39, 40). Nevertheless, their incremental validity, feasibility and effect on meaningful health outcomes require prospective evaluation before they can be considered reliable components of preventive practice. Pilot studies in school settings suggest that image-based tools and digital platforms can support dietary assessment by estimating food intake, portion sizes, and food waste. These tools have potential applications in nutrition education (41, 42). Wearable sensors and mobile technologies could complement these approaches by continuously monitoring physical activity, sedentary behavior and sleep and by providing self-monitoring, feedback and goal-setting functions (43–46). A 2024 systematic review and meta-analysis of step-monitoring interventions reported small reductions in BMI z-score, body fat and body weight among children and adolescents, but no significant effects on BMI or waist circumference; evidence of sustained benefit beyond the active intervention was limited (44). Broader reviews found that paediatric wearables were generally feasible and acceptable but reported heterogeneous effects on physical activity, declining engagement over time, variable measurement accuracy and frequent reliance on caregiver-managed smartphone applications (45, 46). Wearables may therefore reinforce behavior change when embedded in family-, school- or service-based multicomponent programmes, but they should not be presented as stand-alone solutions. Importantly, they cannot offset the unaffordability of healthy foods or other structural barriers to Mediterranean diet adherence. Their public health value will also depend on affordable and equitable access, age-appropriate design, data protection, nutritional literacy and supportive food and physical activity environments, particularly for socioeconomically disadvantaged families.

From a public health perspective, AI applications for tackling childhood obesity can be categorized into four main areas: predicting and identifying risk factors early on; behavioral interventions; precision nutrition; and monitoring of environments that encourage obesity (47–49). Although some predictive models report apparently high discrimination (e.g., concordance indices of 0.769 for obesity and 0.835 for overweight), such performance estimates are not equivalent to clinical or public health benefit, and implementation remains limited by heterogeneity and inadequate external validation (50). Closer examination nevertheless shows that these apparent advances rest on a methodologically immature evidence base. In the prediction-model review, 45 studies yielded 126 models, but high risk of bias was common because many datasets were cross-sectional, development samples were insufficient relative to the number of predictors, missing data were inadequately handled, overfitting occurred, and independent validation cohorts were absent or too small (50). A recent Cochrane review identified only two randomized controlled trials (n = 105) with very low to low certainty of evidence regarding BMI outcomes (51). Only one trial (n = 65) contributed primary outcome data, while imprecision, indirectness, and incomplete reporting of randomisation, blinding, and compliance precluded practice recommendations (51). More broadly, the available AI literature is dominated by pilot, feasibility, and observational studies with limited samples, short follow-up, heterogeneous interventions and outcomes, and scarce evidence on real-world effectiveness, long-term safety, and cost-effectiveness (49). Together, these limitations delineate the gap between technological development and clinical and public health applicability.

Beyond individual-level applications, AI is increasingly being used to assess the structural determinants of obesity. Computer vision and deep learning techniques can identify child-directed food marketing on a large scale. For instance, a convolutional neural network (CNN) model achieved a high level of accuracy (90%) and an area under the curve (AUC) of 96%, revealing that a significant proportion of marketed breakfast cereals are aimed at children and classified as unhealthy according to nutritional standards (48). However, these findings should be interpreted cautiously: the annotated dataset did not encompass the full food supply, performance was lower for abstract or infrequently represented marketing features, and the application was restricted to static packaging and a selected Canadian product category. Moreover, its cross-sectional, observational analyses cannot establish causal relationships between marketing, nutritional quality, and price, while data from 2017–2020 require updating and model retraining to reflect current marketing practices (48).

Extending AI from individual risk prediction to environmental monitoring broadens its potential public health scope, but does not by itself demonstrate that the resulting information will change policy, improve outcomes or reduce inequalities. At the individual level, algorithms may identify risk patterns and tailor feedback; at school and community levels, linked dietary, activity and contextual data may reveal where support is most needed; and at population level, AI-enabled monitoring may map food outlets, prices, product availability and marketing exposure. However, improved measurement and prediction do not themselves change the commercial, economic and material conditions that shape children’s diets. Evidence that proximity to fast-food outlets is associated with obesity, whereas access to supermarkets and fruit and vegetable retailers is inversely associated, reinforces the need for regulatory, retail and urban-planning responses (52). AI-derived evidence can inform action only when it is linked to interventions such as healthy school food procurement, restrictions on unhealthy marketing, measures to improve the affordability and availability of nutritious food, and adequately resourced family and community services. AI-driven personalised nutrition should therefore be understood as a complement to, not a substitute for, structural prevention.

### AI, equity and data governance in childhood obesity

In order to avoid exacerbating existing health disparities, an equity-by-design approach must inform the development and implementation of artificial intelligence (AI) in public health nutrition. Evidence from the World Health Organization Regional Office for Europe highlights persistent gaps between policy intentions and practical implementation, largely due to a lack of standardized equity metrics and limited frameworks for assessing algorithmic bias (53, 54). In this context, AI tools should be evaluated not only on their predictive accuracy, but also on their implications for equity, accessibility, and cultural appropriateness across different population subgroups.

Effective and fair implementation of AI also requires robust data governance. A WHO Europe policy brief (55) emphasizes the importance of high-quality, representative, ethically governed datasets to minimize algorithmic bias, and to support equity, gender equality, and human rights in digital health applications.

### Digital divide and implementation barriers

A key challenge in implementing digital health technologies is the digital divide, which can exacerbate existing social inequalities. In Italy, for example, ISTAT data indicates that there are persistent disparities in internet access related to educational level and geographic location. There is a clear gap between the northern and central regions and the southern areas (56). These inequalities may reduce the effectiveness of digital health interventions and contribute to widening disparities in the prevention and management of childhood obesity (53, 54, 57).

Addressing these challenges requires inclusive support systems adapted to user diversity. Human mediation, multilingual design, affordable access and offline alternatives are needed to avoid widening existing inequalities.

Implementation must also be judged by its distributional effects. A systematic review of 27 adolescent obesity studies found that only eight targeted disadvantaged populations and that 82% of the assessed digital-health equity criteria were not addressed; whether digital interventions reduce or widen inequalities therefore remains uncertain (58). Implementation should monitor reach, engagement and outcomes by socioeconomic position, geography and digital access, and involve underserved adolescents in co-design.

### Limitations and research priorities for AI-enabled interventions

Current evidence does not support AI-enabled personalised nutrition as an established intervention for preventing childhood obesity. Existing studies largely concern prediction, technical performance or feasibility, with limited external validation and little evidence from adequately powered comparative trials (49–51). Improvements in discrimination, dietary recording or engagement should not be equated with sustained changes in diet, BMI trajectories, psychosocial well-being or inequalities, and rapidly changing platforms require continued validation after implementation.

Research should test whether AI adds value beyond established nutritional assessment, professional support and public health programmes, using representative samples, meaningful outcomes, independent validation and sufficient follow-up (50, 51). Equity should be treated as a primary outcome, with transparent subgroup reporting and co-design involving children, families and underserved communities (53, 54, 57, 58). Until such evidence is available, AI should remain a research and decision-support adjunct, supported by human oversight, privacy safeguards and non-digital alternatives.

### Implications for public health policy

The evidence reviewed here supports moving beyond descriptive analyses of childhood obesity toward system-level prevention strategies. In line with the European Commission’s Farm to Fork Strategy (5), policies should prioritize improving access to healthy food and reducing exposure to ultra-processed products and unhealthy food marketing. Equity-oriented approaches, including proportionate universalism, remain central to effective interventions.

In Italy, digital and AI-based tools should be integrated into existing public health and educational systems, including the OKkio alla Salute surveillance program and national guidelines (59). Such integration would help ensure that technological innovation complements rather than replaces established public health structures. Schools and community settings remain key environments for prevention through regulation of the food environment and promotion of the Mediterranean diet. In these settings, digital tools can support surveillance and identify inequalities within multidisciplinary public health action.

Effective childhood obesity prevention ultimately requires multisectoral action within a Health in All Policies framework. Robust governance mechanisms for data protection, transparency, and equity monitoring are essential to ensure that digital innovations contribute to, rather than undermine, public health goals.

## Conclusion

Childhood obesity is the result of an interaction between evolving food systems, dietary habits and social inequalities. Effective prevention strategies should combine sustainable dietary patterns with school- and community-based interventions that are integrated into equity-oriented public health policies. In this context, artificial intelligence and digital tools could support surveillance, the early identification of risks, and targeted prevention, provided they are backed by multisectoral actions integrated into existing healthcare and community systems. Even robust dietary traditions such as the Mediterranean diet require a supportive structural, educational and social environment to be maintained over time. At present, these technologies should be treated as investigational and supportive rather than as established preventive solutions, and their evaluation should prioritize long-term effectiveness, implementation feasibility and equity impact.

More specifically, the principal evidence gap is the distance between algorithm development and intervention evaluation.

The next phase should prioritise evaluation rather than further demonstrations of technical accuracy. Adequately powered multicentre randomised controlled trials and pragmatic cluster trials should test genuinely adaptive AI-enabled programmes against usual care and matched non-AI digital interventions, with sufficient follow-up to examine dietary quality, BMI trajectories, mental and functional well-being, adverse events and maintenance. Prospective external-validation and real-world deployment studies should assess calibration drift, safety, clinician workload, reach, adoption and cost-effectiveness, while hybrid effectiveness-implementation studies should determine how AI can be integrated into schools, primary care and community services.
